# Perceived patient satisfaction with in-patient services at Jimma University Specialized Hospital, Southwest Ethiopia

**DOI:** 10.1186/s13104-015-1179-8

**Published:** 2015-07-01

**Authors:** Tirsit Retta Woldeyohanes, Tewodros Eyob Woldehaimanot, Mirkuzie Woldie Kerie, Mubarek Abera Mengistie, Elias Ali Yesuf

**Affiliations:** Department of Dermatovenereology, College of Public Health and Medical Sciences, Jimma University, Jimma, Ethiopia; Department of Pharmacy, College of Public Health and Medical Sciences, Jimma University, Jimma, Ethiopia; Department of Health Services and Management, College of Public Health and Medical Sciences, Jimma University, Jimma, Ethiopia; Department of Psychiatry, College of Public Health and Medical Sciences, Jimma University, Jimma, Ethiopia

**Keywords:** Satisfaction, Inpatient, Patient, Jimma University Specialized Hospital, Ethiopia

## Abstract

**Background:**

Patient satisfaction is an attitude resulting from a person’s general orientation towards a total experience of health care. It is a key determinant and a legitimate measure for quality of care. In developing countries, satisfaction studies were conducted mainly on nursing care and outpatient services.

**Objective:**

This study aims to measure and describe the level of patient satisfaction within inpatient health care services.

**Methods:**

Across sectional study design was conducted from 8 May 2011 to 2 June 2011 at Jimma University Specialized Hospital. Systematic random sampling technique was employed to recruit participants. A standardized structured questionnaire developed by reviewing similar literatures was used to assess the level of patient satisfaction towards the inpatient services. SPSS version 19 statistical packages were used for data management and analysis.

**Result:**

A total of 189 patients participated. The proportion of overall net patient satisfaction was 117 (61.9%). Majority of the respondents 148 (78.3%) reported that they got the kind of service they anticipated. Cleanliness of the ward 145 (76.7%) and time to get back to home 27 (14.3%) were found to have the highest and the lowest proportion of satisfied respondents, respectively. Patients with no formal education 60 (76.9%) and patients from the rural areas 75 (68.8%) were satisfied higher than those from their counterparts. Patients at medical 22 (61.1%) and ophthalmology 10 (62.5%) wards were less satisfied than patients in other departments.

**Conclusion:**

Nearly two third of the patients were found to be satisfied by the service they received from the hospital. Most of the patients found to be dissatisfied with the nursing, pharmacy and laboratory services, while some others were still dissatisfied with the level of health education, communication and information they received about their illness. Therefore, the hospital administration system should best work on new innovative approach to keep and improve the administrative system, waiting time, hospital stay, hospital accommodation, access for medications and laboratory services to bring patient satisfaction. Nurses and physicians should have to work best to improve health education, communication and understanding between doctors/nurses and patients. Hospital reformation and modern hospital administration system could work best to keep and improve the level of patient satisfaction.

## Background

Patient satisfaction is becoming an emerging health policy all over the world. It is a key determinant of quality of care and an important component of pay-for-performance metrics. Furthermore, patient satisfaction is critical to ensure how well patients do; many research clearly identified a link between patient outcomes and patient satisfaction scores [1–3].

Patient satisfaction is multifaceted and a very challenging outcome to define. It seems easy to understand but hard to define. Satisfaction is not a pre-existing phenomenon waiting to be measured, rather a judgment people made reflecting their experience under specific circumstances. A simple and practical definition of satisfaction would be the degree to which desired goals have been achieved [4].

It is a perception and an attitude that a consumer can have or view towards a total experience of health care. It comprises both cognitive and emotional facets and is influenced by previous experience, expectations and social networks [5]. Patient expectations of care and attitudes towards health care system greatly contribute to satisfaction; other psychosocial factors, including pain and depression, are also known to contribute to patient satisfaction scores [6].

Evaluation of clients satisfaction can address the reliability of services, or the assurance that services are provided in a consistent and dependable manner; the responsiveness of services or the willingness of providers to meet clients need; the courtesy of providers; and the security of services and records to keep the best level of confidentiality [7]. Measurement of patient satisfaction plays an important role in the growing push toward accountability among health care providers. Studies on patient satisfaction have a significant role in developing and delivering high quality health care in the hospital with the involvement of patients in the management of their problem and treatment [4, 8, 9].

Therefore, evaluation of healthcare provision is essential to improve and keep the quality of medical services. Traditionally, reports of patients were given less attention compared to technical and functional reports of outcome. More recently, however, healthcare systems have sought to pay attention for acceptability and preference of patients in the hospital [10–12]. The future of patient satisfaction lies in continuing to enhance the environment where people work and where patients receive care [13]. This study is aimed at assessing the level of perceived patient satisfaction with in-patient services at Jimma University Specialized Hospital, Southwest Ethiopia. This study contributes an important understanding to the level of patient satisfaction to the inpatient health care services and fills the knowledge gap to improve service quality.

## Methods

*Study setting* This study was conducted from 8th May to 2nd June 2011 at Jimma University Specialized Hospital (JUSH), located in Jimma town 352 km southwest of Addis Ababa. It is one of the oldest public hospitals in the country established in 1930 E.C by the Italians for the service of their soldiers and used to be named as St. Mary Hospital. Currently it is a teaching hospital with a bed capacity of around 432 with a total of nearly 1,000 hospital staffs. It provides services for approximately 9,632 inpatients, 5,000 accident and emergency cases, and 80,000 outpatient attendants each year [14]. All admitted patients were included in the study during the study period from 8th May to 2nd June 2011.

*Study design* A cross sectional study design was employed.

## Exclusion criteria

Those seriously ill, laboring mothers, and psychiatric and pediatric patients without an attendant were excluded from the study.

## Sample size and sampling procedures

Sample size (n) was determined using single population proportion formula based on the assumption of 77% (unpublished) prevalence, expected margin of error (d) 0.05 at 95% confidence interval (Z_α/2_). After correction the formula (N_adj_ = n/1 + n/N) was employed to adjust for the total population of <10,000, the final sample size became 189. Then the sample size was proportionally allocated to each ward. Systematic random sampling method was used to select of study subjects from the total patients.

### Measurements

Two set of standardized structured questionnaires were developed for the purpose of data collection by reviewing relevant literatures [15–20].

The first questionnaire was composed of basic socio-demographic variables including age, sex, residence, educational, and occupational status. The second included questions about the level of perceived patient satisfaction which was composed of dimensions to measure satisfaction from providers’ side, facility and quality of care on the inpatient health care service. The initial English version of the questionnaire was translated to Amharic and then back-translated into English independently to check for consistency and semantic validity. Each item was scored on a 5 point Likert-scale, ranging from 1 (very satisfied), 2 (satisfied), 3 (neutral), 4 (dissatisfied)and 5 (very dissatisfied). The mean score of satisfaction for each patient was calculated as the average of satisfaction items. A mean score of 3 or more were taken as an indicator of patient’s perceived dissatisfaction. Score 3 (neutral) was considered as dissatisfied because patients may be afraid to state their dissatisfaction of the services they were receiving. The overall patient satisfaction was a measured value using one item in the questionnaire stating “How do you rate your overall level of satisfaction regarding the health service you received in this hospital?” Net overall or net satisfaction was a calculated value and refers to the proportion of patients whose mean score of satisfaction was <3.

### Data collection process

The patients were interviewed using the structured questionnaire. The questionnaire obtained information on socio demographic characteristics of the respondents and their level of satisfaction with the hospital services including the availability of drugs and supplies, information provision by the health workers, waiting time to get the services, courtesy and respect of the health workers, and cleanness of the wards. The data was collected by six medical interns.

### Data quality assurance

After training of the data collectors, the questionnaire was pretested to ensure the acceptability, comprehensibility and understandability by the participants. Regular supervision, spot checking and reviewing the completed questionnaire was carried out daily by the principal investigator. Data was checked and entered into a computer. All the entered data were checked before final analysis.

### Data processing and analysis

SPSS version 19 statistical package was used for data management and analysis. Before final analyses the principal investigator performed data cleaning by looking at the distribution of the data, identifying outliers and checking back against the original data. Most of the responses were analyzed descriptively with simple frequency distribution and percentages as a measure of central tendency. Chi square (chi-^2^) test was performed to detect associations at 5% level of significance for selected variables.

### Ethical consideration

Approval for the ethical clearance was obtained from the ethical review board of College of Public Health and Medical Sciences (CPHMS). Written informed consent was obtained from each participant and they were also informed that they have the right to withdraw from the study at any point in time. Issue of privacy and confidentiality were strictly maintained.

## Results

### Socio-demographic

A total of 189 patients were participated making a response rate of 100%. Of the total participants, 102 (54%) were females and 119 (63%) were married. The mean age and standard deviation (SD) of the participants were 26.53 ± 15.10 years. In terms of religious affiliation, 95 (50.3%) of the patients were Muslims and [63 (33.3%)] were Orthodox Christians. Regarding to ethnicity, 104 (55%) of the patients were Oromo while 25 (13.2%) were Amhara. About 118 (62.4%) of the patients stayed in the hospital for more than 3 days. The mean hospital stay was 2.55 ± 0.64 days (Table 1).

**Table 1:** Socio-demographic characteristics of respondents (n = 189)

Variables	Category	n (%)
Sex	Male	87 (46.0)
	Female	102 (54.0)
Age (in years)	<15	43 (22.8)
	15–19	16 (8.5)
	20–24	34 (18.0)
	25–29	30 (15.9)
	30–34	17 (9.0)
	35–39	13 (6.9)
	40–44	8 (4.2)
	45–49	8 (4.2)
	50–54	8 (4.2)
	55–59	7 (3.7)
	≥60	5 (2.6)
Marital Status	Single	43 (22.8)
	Married	119 (63.0)
	Divorced	17 (9.0)
	Widowed	10 (5.3)
Living status	Alone	28 (14.8)
	Live with others	161 (85.2)
Educational Status	No formal education	78 (41.3)
	Primary school	35 (18.5)
	Secondary school	39 (20.6)
	Preparatory [11, 12]	5 (2.6)
	Vocational or certificate	13 (6.9)
	Diploma and above	19 (10.1)
Occupational status	Government employee	20 (10.6)
	Merchant	12 (6.3)
	Farmer	74 (39.2)
	Jobless	21 (11.1)
	Student	26 (13.8)
	Others	36 (19.0)
Residence	Urban	80 (42.3)
	Rural	109 (57.7)
Payment status	Paying	64 (33.9)
	Free	125 (66.1)
Frequency of visit	New visit	144 (76.2)
	Repeat visit	45 (23.8)
Religion	Muslim	95 (50.3)
	Orthodox	63 (33.3)
	Protestant	30 (15.9)
	Others	1 (0.5)
Ethnicity	Oromo	104 (55.0)
	Amhara	25 (13.2)
	Keffa	22 (11.6)
	Gurage	15 (7.9)
	Tigre	7 (3.7)
	Others	16 (8.5)
Hospital stay	<1 day	15 (7.9)
	1–3 days	56 (29.6)
	>3 days	118 (62.4)
Ward	Medical	36 (19.0)
	Surgical	50 (26.5)
	Pediatrics	40 (21.2)
	Maternity	17 (9.0)
	Gynecology	18 (9.5)
	Psychiatry	12 (6.3)
	Ophthalmology	16 (8.5)

### Inpatient satisfaction on health care services at JUSH

The overall and net overall satisfaction of patients admitted in the hospital were 127 (67.2%) and 117 (61.9%), respectively. Majority of the respondents 148 (78.3%) reported that they received the kind of service that they anticipated from the hospital. Most 116 (61.4%) of the patients got a bed within a day with a mean time of 1.53 ± 0.74 days. Nearly all patients 175 (92.6%) had received laboratory and x-ray request; of whom only 59 (33.7%) of the patients got all the requested services while 98 (56%) of the patients got only some of the requested services from the hospital they were admitted. About 18 (10.3%) of the patients got none of the requested laboratory services from the hospital. Regarding the time to give laboratory specimen to the laboratory technologist, most 139 (88.5%) gave within an hour with an average time of 1.11 ± 0.32 h. About 134 (85.4%)of the patients received their laboratory result within 1–12 h with a mean waiting time of 1.87 ± 0.36 h. Out of the 70 x-rayed patients, 26 (37.1%) took 1–2 h to be x-rayed with a mean time of 1.91 ± 0.79 h. Most 92 (52.6%) patients got a physician within an hour after receiving their laboratory result. About 161 (85.2%) patients had received prescription paper for medication of whom only 62 (38.5%) of the patients had all the drugs from inpatient pharmacy.

Majority 168 (88.9%) of the patients were able to communicate with nurses and physicians without any barriers of communication and 100 (52.9%) of the respondents had received medical help at night at the time they need it. The remaining 21 (11.1%) patients were unable to communicate with the nurses and physicians due to language barrier and almost all 20 (95.2%) of them were dissatisfied with the absence of interpreter service. Most 183 (96.8%) patients felt that they are safe in the hospital and 160 (84.7%) of the patients said that they would tell others to use this hospital while 162 (85.7%) of the patients said they might return back for treatment.

In all inpatient health care services, “cleanliness of the ward” was scored the highest 145 (76.7%) proportion of satisfaction while the recommended time to get back home had the highest 162 (85.7%) proportion of dissatisfaction. Patients were also satisfied with the admission service, waiting time, physician skill, whereas dissatisfaction level was significantly higher for information service of the hospital, nursing service, illness education/communication, privacy and confidentiality, completeness of the information given, crowded rooms, dietary services, visiting hours, and services to pharmacy and laboratory (Table 2).

**Table 2:** Level of satisfaction of respondents with the different components of health care services (n = 189)

Items	n (%)	
	Satisfied	Dissatisfied
Information on the services of the hospital	80 (42.3)	109 (57.7)
Satisfaction with the admitting service	109 (57.7)	80 (42.3)
Satisfaction with the waiting time	138 (73)	51 (27.0)
Satisfaction with the nursing service	94 (49.7)	95 (50.3)
Satisfaction with the physician service	114 (60.3)	75 (39.7)
Satisfaction with health education	71 (37.6)	118 (62.4)
Privacy	38 (20.1)	151 (79.9)
Toilet cleanliness	35 (18.5)	154 (81.5)
Time to get back home (hospital stay)	27 (14.3)	162 (85.7)
Availability and drug supply satisfaction in the inpatient pharmacy	87 (54.7)	72 (45.3)
Completeness of the information given	57 (30.2)	132 (69.8)
Measures taken to assure confidentiality	46 (24.3)	143 (75.7)
Ward cleanliness	145 (76.7)	44 (23.3)
Room accommodation	57 (30.2)	132 (69.8)
Bed cleanliness	68 (56.2)	121 (43.8)
Dietary service	84 (44.4)	105 (55.6)
Visiting hours	49 (25.9)	140 (74.1)
Way questions and queries dealt by staff	75 (39.7)	114 (60.3)
Outpatient pharmacy satisfaction	17 (15.6)	92 (84.4)
Satisfaction on the laboratory	54 (30.9)	121 (69.1)
Overall satisfaction	127 (67.2)	62 (32.8)
Overall net/net satisfaction	117 (61.9)	72 (38.1)

Level of patients’ educational status and address were found to have significant association with the level of net patients’ satisfaction. It was observed that patients with no formal education were more satisfied 60 (76.9%) than their counterparts (x^2^ = 17.006, p = 0.004); and also patients from urban areas were less 42 (52.5%) satisfied than those from the rural area (X^2^ = 5.203, p = 0.023) (Table 3).

**Table 3:** Comparison of net satisfaction by Sociodemographic characteristics (n = 189)

Variables	Categories	n (%)		Chi square	*p* value
		Satisfied	Dissatisfied		
Sex	Male	53 (60.9)	34 (39.1)	0.066	0.797
	Female	64 (62.7)	38 (37.3)		
Age (in years)	<15	31 (72.1)	12 (27.9)	10.272	0.417
	15–19	9 (56.2)	7 (43.8)		
	20–24	17 (50.0)	17 (50.0)		
	25–29	17 (56.7)	13 (43.3)		
	30–34	11 (64.7)	6 (35.3)		
	35–39	10 (76.9)	3 (23.1)		
	40–44	4 (50.0)	4 (50.0)		
	45–49	7 (87.5)	1 (12.5)		
	50–54	4 (50.0)	4 (50.0)		
	55–59	5 (71.4)	2 (28.6)		
	≥60	2 (40.0)	3 (60.0)		
Marital status	Single	23 (53.5)	20 (46.5)	2.045	0.563
	Married	78 (65.5)	41 (34.5)		
	Divorced	10 (58.8)	7 (41.2)		
	Widowed	6 (60.0)	4 (40.0)		
Living status	Alone	17 (60.7)	11 (39.3)	0.020	0.888
	Live with others	100 (62.1)	61 (37.9)		
Educational status	No formal education	60 (76.9)	18 (23.1)	17.006	0.004
	Primary school	18 (51.4)	17 (48.6)		
	Secondary school	23 (59.0)	16 (41.0)		
	Preparatory [11, 12]	3 (60.0)	2 (40.0)		
	Vocational or certificate	7 (53.8)	6 (46.2)		
	Diploma and above	6 (31.6)	13 (68.4)		
Occupational status	Farmer	51 (68.9)	23 (31.1)	5.152	0.398
	Merchant	9 (75.0)	3 (25.0)		
	Government employee	10 (50.0)	10 (50.0)		
	Jobless	11 (52.4)	10 (47.6)		
	Student	14 (53.8)	12 (46.2)		
	Others	22 (61.1)	14 (38.9)		
Residence	Urban	42 (52.5)	38 (47.5)	5.203	0.023
	Rural	75 (68.8)	34 (31.2)		
Payment status	Paying	37 (57.8)	27 (42.2)	0.687	0.407
	Free	80 (64.0)	45 (36.0)		
Frequency of visit	New visit	92 (63.9)	52 (36.1)	1.010	0.315
	Repeat visit	25 (55.6)	20 (44.4)		
Religion	Muslim	68 (71.6)	27 (28.4)	8.753	0.033
	Orthodox	33 (52.4)	30 (47.6)		
	Protestant	16 (53.3)	14 (46.7)		
	Others	0 (0)	1 (100)		
Ethnicity	Oromo	76 (73.1)	28 (26.9)	20.782	0.001
	Amhara	17 (68.0)	8 (32.0)		
	Keffa	10 (45.5)	12 (54.5)		
	Gurage	6 (40.0)	9 (60.0)		
	Tigre	4 (57.1)	3 (42.9)		
	others	4 (25.0)	12 (75.0)		
Hospital stay	<1 day	9 (60.0)	6 (40.0)	1.198	0.549
	1–3 days	38 (67.9)	18 (32.1)		
	>3 days	70 (59.3)	48 (40.7)		
Ward	Medical	14 (38.9)	22 (61.1)	17.241	0.008
	Surgical	34 (68.0)	16 (32.0)		
	Pediatrics	28 (70.0)	12 (30.0)		
	Maternity	13 (76.5)	4 (23.5)		
	Gynecology	13 (72.2)	5 (27.8)		
	Psychiatry	9 (75.0)	3 (25.0)		
	Ophthalmology	6 (37.5)	10 (62.5)		

Patients at medical and ophthalmology wards were also less satisfied than patients admitted to other wards (X^2^ = 17.241, p = 0.008). Patients whose waiting time were <1 day were more 83 (71.6%) satisfied than those patients whose waiting time was more than one day (X^2^ = 12.078, p = 0.002). No association was found between sex, age, marital status, living and occupational status, frequency of visit and hospital stay to level of patient satisfaction (Table 4).

**Table 4:** Comparison of satisfaction by time related variables (n = varies for each service)

Time taken to	Durations	n (%)		Chi square	p value
		Satisfied	Dissatisfied		
Get bed (n = 189)	<1 day	83 (71.6)	33 (28.4)	12.078	0.002
	1–3 days	20 (44.4)	25 (55.6)		
	>3 days	14 (50.0)	14 (50.0)		
See the admitting staff (n = 189)	<1 h	95 (65.1)	51 (34.9)	2.749	0.253
	1–2 h	11 (50.0)	11 (50.0)		
	>2 h	11 (52.4)	10 (47.6)		
Be X-rayed (n = 70)	<1 h	16 (64.0)	9 (36.0)	2.440	0.295
	1–2 h	16 (61.5)	10 (38.5)		
	>2 h	8 (42.1)	11 (57.9)		
Give laboratory specimen (n = 157)	<1 h	88 (63.3)	51 (36.7)	0.033	0.856
	1–12 h	11 (61.1)	7 (38.9)		
Receive laboratory result (n = 157)	<1 h	16 (72.7)	6 (27.3)	1.669	0.434
	1–12 h	82 (61.2)	52 (38.8)		
	>12 h	1 (100)	0 (0)		
See physician after diagnostics (n = 174)	<1 h	63 (68.5)	29 (31.5)	3.220	0.359
	1–12 h	46 (59.7)	31 (40.3)		
	>12 h	2 (40.0)	3 (60.3)		

## Discussion

In general this study found out that the proportion of overall net patient satisfaction was 117 (61.9%). Majority of the respondents 148 (78.3%) reported that they received the kind of service they anticipated. In this study the level of net satisfaction about medical services was 61.9% which was much lower compared to a study conducted in Bangkok 91.7% [21]. However it is higher compared to the study conducted in Mozambique 55% [22]. The possible explanation of this difference could be due to Thailand being somehow a developed country with better quality of health care system than Ethiopia and also the study had included observation and inclusion of hospital staff in their study.

Though cleanliness of the ward was widely endorsed item by the patients (76.6%) as the highest rate of satisfaction in this study, it is still low when compared to a study in Dareselam and Tanzania which was 88.5% [23]. This study showed that the highest 85.7% rate of dissatisfaction was due to the recommended time to get back home which could be due to the fact that most patients stayed in the ward for more than 3 days while attendants are also expected to care for other members of the family at home.

Regarding the service provided by the physician, 60.3% of the patients were satisfied with their knowledge, courtesy and respect to them. But it was still low when compared to a report from a study conducted in Singapore hospitals, where “Doctors explain thoroughly about the medical conditions the patients having” [24]. But 62.4% of the patients were dissatisfied with the level of education and communication they received about their illness and 69.8% of patients had received incomplete information about their illness which is higher compared to a study conducted in South Africa [25, 26]. This difference could be explained by high number of patients expected to be seen by a physician resulting in shortage of time and also due to low level of education of the patients which might be a barrier in understanding of communication.

More than 54.7% of patients were satisfied by the drug availability and supply in the inpatient pharmacy which was much higher compared to that of a study in Tigrai Zonal hospitals which reported that nearly 34% of the clients were nonpaying and about 61% of those clients with prescription paper for drugs did not get the ordered drugs from the hospital pharmacies [27]. However, the proportion of satisfied patients due to medication availability (54.7%) were lower compared to a study done in hospitals of Amhara region in which about 66% of the clients obtained the prescribed drugs [28].

About 56.2% of the patients were also satisfied with the cleanliness of the bed which is low as compared to a study in Tanzania 72.8% [23] which could be explained by unrealistic expectation by patients from rural area who have no prior exposure for hospital environment in the nearby. Nearly 57.7% of patients were dissatisfied with the provision of information on the services of which is high compared to the study conducted in Tigrai zonal hospitals with 46.7% dissatisfaction rate [27]. This could be due to the recently implemented government policy Business Process Re-engineering (BPR) to transform the health care system and increased number of health professionals at Tigray Zonal Hospital.

Most of the dissatisfaction scores were higher for nursing services, education and communication about the illness, privacy and confidentiality, crowdedness of rooms, restricted visiting hours, outpatient pharmacy and laboratory services. The reason for this could be that most of these services are labor intensive while some of the other services like X-ray and laboratory services are financially coasty.

In related literatures, studies that analyze the influence of socio-demographic characteristics on patients’ satisfaction showed no consistent relationship with age, race, gender, education or income. Some previous studies, however, have found that older patients were more likely to report satisfaction compared with younger patients. In some studies, females were more likely to express satisfaction than males while in others no relationship was found between gender and satisfaction [29–32]. In this study however, there was no observed association between satisfaction and basic socio-demographic variables.

### Limitations of the study

Since patients were interviewed in the hospital setting, they may give responses favoring the care provider resulting in social desirability bias.

## Conclusion

Based on the findings of this descriptive cross sectional study, nearly two third of the patients have showed overall satisfaction. The level of satisfaction was found to be significantly affected by patient educational status, address, ward and the waiting time to get bed.

### Recommendations

The hospital administration system should best work on new innovative approach to keep and improve the administrative system, waiting time, hospital stay, hospital accommodation, access for medications and laboratory service to bring patient satisfaction. Nurses and physicians should have to work best to improve health education, communication and understanding between doctors/nurses and patients. Hospital reformation and modern hospital administration system could work best to keep and improve the level of patient satisfaction.
